# Erosion of Trust in the Medical Profession in India: Time for Doctors to Act

**DOI:** 10.15171/ijhpm.2016.143

**Published:** 2016-11-02

**Authors:** Sumit Kane, Michael Calnan

**Affiliations:** ^1^KIT Health, Royal Tropical Institute, Amsterdam, The Netherlands.; ^2^Gokhale Institute of Politics and Economics, Pune, India.; ^3^School of Social Policy and Social Research, University of Kent, Canterbury, UK.

**Keywords:** Trust, Stewardship, Regulation, Health System, India

## Abstract

In India, over the last decade, a series of stewardship failures in the health system, particularly in the medical profession, have led to a massive erosion of trust in these institutions. In many low- and middle-income countries (LMICs), the situation is similar and has reached crisis proportions; this crisis requires urgent attention. This paper draws on the insights from the recent developments in India, to argue that a purely control-based regulatory response to this crisis in the medical profession, as is being currently envisaged by the Parliament and the Supreme Court of India, runs the risk of undermining the trusting interpersonal relations between doctors and their patients. A more balanced approach which takes into account the differences between system and interpersonal forms of trust and distrust is warranted. Such an approach should on one hand strongly regulate the institutions mandated with the stewardship and qualities of care functions, and simultaneously on the other hand, initiate measures to nurture the trusting interpersonal relations between doctors and patients. The paper concludes by calling for doctors, and those mandated with the stewardship of the profession, to individually and collectively, critically self-reflect upon the state of their profession, its priorities and its future direction.

Over the last few years, the medical profession in India has been in a protracted state of crisis. Doctors across the country have been exposed and indicted on counts of corruption, professional negligence, taking kickbacks, and illegal dual practice, both in the court of law, and in society at large.^1-4^ The statutory body responsible for stewardship of the medical profession is the Medical Council of India (MCI)^5^; its mandate is to oversee medical education, professional and ethical standards in the medical profession, and the registration of medical doctors in India. With multiple and ever serious allegations and indictments related to corruption, incompetence and dereliction of duties in checking the misconduct amongst doctors, the MCI is at the heart of this crisis.^6-9^ In a dramatic turn of events, a recent Parliamentary Committee report on the functioning of the MCI noted that “the Medical Council of India … has repeatedly failed on all its mandates over the decades,” and that the state of the medical profession is perhaps at its “lowest ebb” (p.20).^10^ In an exceptional move, on May 2, 2016, the Supreme Court of India also intervened using its rare and extraordinary powers under the Constitution, to set up a three-member committee headed by a former chief justice of India, to oversee the process of overhauling of the regulatory framework of the medical profession.^11^ In their judgment, the Supreme Court of India, added “that the need for major institutional changes in the regulatory oversight of the medical profession in the country is so urgent that it cannot be deferred any longer.” The parliamentary committee tellingly added that “respect for the profession has dwindled and distrust replaced the high status the doctor once enjoyed in society” (p.110).^8^ This erosion of trust is not a problem that is unique to the medical profession in India; evidence shows that it is a growing concern, globally,^12,13^ and the Indian situation has parallels in many low- and middle-income country (LMIC) health systems.^14-16^ A critical analysis of India’s response to the situation it faces can provide useful insight not only for India, but also to policy-makers in other LMIC contexts; this is the purpose of this paper.

To better understand this erosion of trust, it is important to understand and unpack the social phenomenon of ‘trust’ in the healthcare context. Trust is particularly important in the context of healthcare because it is a means of bridging the vulnerability, uncertainty and unpredictability inherent to the provision of healthcare.^17^ Relationships of trust involve one party, the trustor, harbouring positive expectations regarding the competence of the other party, the trustee (competence trust), and also the trustor, harbouring an expectation that the trustee will work in his/her best interest (intentional trust).^16^ It has been argued that a more earned and conditional or critical trust is an appropriate basis for the doctor-patient relationship.^16^ This is considered appropriate because of both, the costs and dangers of blind trust wherein there is a risk of corruption, exploitation, or domination particularly for those with a lack of resources, as well as due to the imperatives related to patient autonomy preferences.^18^ Another important way of understanding trust relations in the context of healthcare is to distinguish between interpersonal trust – the trust between individual patient and individual care provider/doctor, and institutional trust, which relates primarily to trust in the medical profession or in the healthcare system. Some authors refer to the latter as systems trust, which signifies “accountability and the checks and balances and systems that maintain fairness, preventing incompetence or malign intent”(p. 9).^19^ How systems trust and interpersonal trust relate to each other is, however, quite complex; trust in a particular care provider does not necessarily translate into trust in the medical profession or in the system as a whole, or vice versa.^16^ Finally, a key feature of trust as a relational construct is its fragility; while it is difficult to earn trust, it is easy to lose it; trust needs to be continuously earned to maintain it at an optimal level, and to allow the doctor-patient relationships to function well.^20^ It is with these understandings of the concept of trust that this paper argues for a more nuanced analysis of the state of affairs in the medical profession and the responses to it, in India. It is contended here that reflecting on the situation in India and its responses to the situation, can provide meaningful insights for medical professionals and policy-makers grappling with similar situations in other parts of the world.

The failures of stewardship of the medical profession by the MCI in India have led to erosion of systems trust in the medical profession, but assuming and equating this to be an equal erosion of trust in the interpersonal relationships between individual patients and their doctors, is both, inaccurate, and unhelpful. While large scale survey data is not available from India, evidence from other parts of the world,^21^ and from the few studies on the subject from India^22-24^ bears out that while there may be a decline in trust in the medical profession or in the various institution of the healthcare system, the levels of trust between patients and individual providers may still remain very high. Patients may be more likely to have a trusting relationship in the doctor that ‘they know’ compared with more generalized trust in the medical profession as an institution which may be based less on direct experience and more on second-hand reports, such as those framed through the media.

The intervention by the Supreme Court of India has been lauded, but the response envisaged has also been criticized for not sufficiently taking into account the politics and the risks related to capture of the response by vested interests.^25^ Our argument here is that this response is also problematic as it does not sufficiently distinguish between the erosion of trust in the institutions mandated with the stewardship of the medical profession, the so called systems trust, from the interpersonal trust between individual providers and their patients; we argue that not doing so runs the risk of doing harm and undermining the provider-patient relations. Both, the Parliamentary Standing Committee, and the Supreme Court of India, take a highly legalistic and normatively judgmental view – and approach the whole matter as a regulatory problem; they throughout argue that the medical profession is out of control, and needs to be controlled more effectively. A ‘control’ heavy approach might be beneficial in most other sectors, but in the healthcare sector, it is worth thoroughly examining the duality of trust and control, before moving further. A control-based approach works when and if the person’s positive expectations are based solely on the structural influences shaping the actions of the other,^26^ as is the case of people’s relations with institutions and expert systems like the medical profession. However, when a person’s positive expectations are based, not only on the structural influences shaping the actions of the other, but also on an assumption of benevolent agency or altruistic motives on the part of the other, as is the case in the doctor-patient interpersonal relationship, a trust-based approach, and not a control-based approach, is more appropriate.^16^

A radical control-based regime to address what ails the medical profession in India could be an effective approach for rebuilding trust in the MCI, but applying the same treatment regime to the interpersonal relations between the patient and her doctor, would be inappropriate and akin to making a wrong diagnosis and also giving the incorrect treatment. The English National Health Service’s (NHS’s) experience with the so-called new public management approach, provides valuable insight. It shows that a command and control approach with its emphasis on performance management or the incentivising control approach with its emphasis on choice and competition, adopted in the early part of the century, had limited success, not least because it appeared to have an adverse effect on interpersonal trust between doctors and patients.^27^ The checking-based ‘audit culture’ that accompanied such an approach, and which relied upon crude targets and measures that did not reflect important aspects affecting patient outcomes, could not do justice to the meaning, complexity and specificities inherent to doctors’ work, particularly the relational aspects; it, thus, failed to command legitimacy and credibility amongst professionals. On the contrary, this approach in NHS England, with its focus on control of competence, and neglect of the relational and intentional aspects of the doctor-patient relationship, created a culture of low trust, particularly at the expense of the altruistic intentions and social motives of the doctors.^27^ This leads these authors to argue that policy responses need, therefore, to focus both on competence and intentional trust; the latter tends to be enhanced by relational aspects of trust. We argue for an approach to trust building which emphasises reflection and mutual learning based on conditional and earned trust, but which also respects the specialist expertise of the medical professionals.^28^ Thus, the Supreme Court of India, as it goes about doing this important work, should ensure that the professional norms, altruistic intentions, moral agency and social motives of doctors are not crowded out,^29^ and thereby the interpersonal trust between patients and their doctors is not undermined, by the control measures it recommends to tackle the failures in the stewardship of the medical profession.

The current state of evidence on interventions to improve trust in doctors, is inconclusive.^30^ However, based on the existing evidence, both from the medical field,^16,30^ but also from broader organizational studies,^31^ a two pronged, collaborative and pragmatic approach is warranted. On one hand, robust and fair control-based interventions which improve the transparency, accountability, performance, and oversight of the functioning of the MCI, and of the professional practice setting, are urgently required. In parallel, context specific initiatives are required which encourage and maintain the trusting interpersonal relations between patients and their providers. Both, the report of the Parliamentary Committee, and the judgment of the Supreme Court of India, appear to paint the whole medical fraternity in India with the same brush, and deem the profession incapable of treating what ails it. While the indictment of the MCI is indeed deserved, painting the whole fraternity with the same brush is not reasonable. We argue for an approach which leverages the professional norms, moral agency and the social motives of the vast majority of doctors, and where doctors are engaged as active partners in bringing about change. Such an approach which steers doctors to collectively, and individually, reflect upon their professional conduct, practices, and standards will allow one to harness “this power of the social in driving behavioural modification via a ‘civilizing process,’ where norms and values compel an enlightened form of self-aware, communicative, and reflexivity towards learning and action.”^29^ Such an approach needs to be based on available evidence,^16^ be developed locally by those to whom it would apply, be tailored to the local context, be locally accountable, and should span across all domains of the medical profession – medical education, private practice, public service, continuous professional development, and care settings. Examples of possible interventions based on current evidence^16,30,31^ include (*i*) Incorporation of innovative and experiential learning-based approaches for delivering medical ethics and medical humanities courses; (*ii*) Development and streamlining of neutral and transparent procedures for recording and resolving medical disputes; (*iii*) Incorporation of professional self-reflection skill development as part of medical and continuous professional education, and create fora for doctors to freely self-reflect in their professional lives; (*iv*) Development and promotion of dialogical processes involving neutral third parties to redress grievances; and (*v*) Development and establishment of non-punitive systems for reporting of medical errors and incidents in private and public facilities.

In conclusion, it is high time that the doctors in India individually and collectively, seriously reflect upon the state of their profession, its priorities and its future direction. Today, a self-administered, long, and structured course of critical self-reflection is the self-prescription, the medical profession needs, both in India, and in many other countries. It is not just the need of the moment, the doctors owe it to their patients.

## Ethical issues

Not applicable.

## Competing interests

Authors declare that they have no competing interests.

## Authors’ contributions

SK conceptualized, drafted and finalized the manuscript. MC reviewed and revised the manuscript.

## Authors’ affiliations

^1^KIT Health, Royal Tropical Institute, Amsterdam, The Netherlands. ^2^Gokhale Institute of Politics and Economics, Pune, India. ^3^School of Social Policy and Social Research, University of Kent, Canterbury, UK.

## References

[1] Jesani A (2014). Professional codes, dual loyalties and the spotlight on corruption. Indian J Med Ethics.

[2] Sachan D (2013). Tackling corruption in Indian medicine. Lancet.

[3] Jain A, Nundy S, Abbasi K (2014). Corruption: medicine’s dirty open secret. BMJ.

[4] Bawaskar HS (2013). The medical trade. Indian J Med Ethics.

[5] Medical Council of India website. http://www.mciindia.org/. Accessed October 26, 2016.

[6] Chatterjee P (2010). Trouble at the Medical Council of India. Lancet.

[7] Tiwari SS (2015). Reforming the medical council of India. Indian J Med Ethics.

[8] Vijayakumar K, Saini N (2013). Medical Council of India in a constitutional crisis. J Indian Med Assoc.

[9] Mani MK (1996). Our watchdog sleeps, and will not be awakened. Issues Med Ethics.

[10] The functioning of Medical Council of India. Department-related parliamentary standing committee on health and family welfare (Report No. 92). India: Parliament of India; March 2016.

[11] Sikri AK. Judgment of the constitutional bench of the supreme court of India, Civil Appeal No. 4060 of Modern dental college and research centre and others versus State of Madhya Pradesh and others. Published May 2, 2016.

[12] Blendon RJ, Benson JM (2014). Public trust in physicians – US medicine in international perspective. N Engl J Med.

[13] Timmermans S, Oh H (2010). The continued social transformation of the medical profession. J Health Soc Behav.

[14] Okello DRO, Gilson L (2015). Exploring the influence of trust relationships on motivation in the health sector: a systematic review. Hum Resour Health.

[15] Tucker JD, Cheng Y, Wong B (2015). Patient-physician mistrust and violence against physicians in Guangdong Province, China: a qualitative study. BMJ Open.

[16] Tucker JD, Wong B, Nie J, Kleinman A (2016). Rebuilding patient–physician trust in China. Lancet.

[17] Calnan M, Rowe R. Trust Matters in Health Care. Berkshire: Open University Press; 2008.

[18] Lee YY, Lin JL (2009). Trust but verify: the interactive effects of trust and autonomy preferences on health outcomes. Health Care Anal.

[19] Pilgrim D, Tomasini F, Yassilev I. Examining trust in health care: a multidisciplinary perspective. Palgrave Macmillan; 2011.

[20] Bruhn JG. Trust and the Health of Organizations. Springer Science; 2001.

[21] Van de Walle S, Marien S (2015). Choice in public health services: a multilevel analysis of perceived primary care doctor choice in 22 countries. Adm Soc.

[22] Gopichandran V, Chetlapalli SK (2013). Factors influencing trust in doctors: a community segmentation strategy for quality improvement in healthcare. BMJ Open.

[23] Baidya M, Gopichandran V, Kosalram K (2014). Patient-physician trust among adults of rural Tamil Nadu: a community-based survey. J Postgrad Med.

[24] Kane S, Calnan M, Radkar A (2015). Trust and trust relations from the providers perspective: the case of the health care system in India. Indian J Med Ethics.

[25] Nagral N, Jain A, Nundy S (2016). A radical prescription for the Medical Council of India: hope for a cure. BMJ.

[26] Mollering G (2005). The Trust/Control Duality: an integrative perspective on positive expectations of others. Int Sociol.

[27] Brown P, Calnan M (2011). The civilizing process of trust: developing quality mechanisms which are local, professional-led and thus legitimate. Soc Policy Adm.

[28] Brown P, Calnan M (2010). The risks of managing uncertainty: the limitations of governance and choice, and the potential for trust. Soc Policy Soc.

[29] Frey BS, Jegen R (2001). Motivation crowding theory: a survey of empirical evidence. J Econ Surv.

[30] Rolfe A, Cash-Gibson L, Car J, Sheikh A, McKinstry B (2006). Interventions for improving patients’ trust in doctors and groups of doctors. Cochrane Database Syst Rev.

[31] Bachman R, Gillespie N, Priem R (2015). Repairing trust in organizations and institutions: Toward a conceptual framework. Organ Stud.

